# New Advances in Diagnostic Radiology for Ischemic Stroke

**DOI:** 10.3390/jcm12196375

**Published:** 2023-10-06

**Authors:** Gabriel Broocks, Lukas Meyer

**Affiliations:** Department of Diagnostic and Interventional Neuroradiology, University Medical Center Hamburg-Eppendorf, Martinistraße 52, 20246 Hamburg, Germany; lu.meyer@uke.de

## 1. Introduction

Ischemic stroke, a leading cause of disability and mortality worldwide, occurs due to the sudden interruption of blood supply to a specific region of the brain. Timely and accurate diagnosis is crucial for providing effective treatment and improved patient outcomes. In recent years, diagnostic neuroimaging has witnessed significant advancements that revolutionized the way ischemic stroke is diagnosed, leading to improved patient care and outcomes. This editorial explores the recent advances in radiology techniques for diagnosing ischemic stroke and highlights their potential to enhance early detection, accurate characterization, and treatment decision making.

## 2. Advances in Stroke Neuroimaging and Treatment

### 2.1. Computed Tomography

Dual-energy CT (DECT), spectral CT, and photon-counting CT are innovative technologies that hold significant promise for enhancing the imaging triage of acute ischemic stroke. These advancements offer improved insights into stroke pathology, leading to more accurate and rapid diagnosis and treatment decisions. DECT employs two different X-ray energy levels, enabling better tissue differentiation and characterization. In stroke cases, this method can distinguish between ischemic and hemorrhagic strokes, resulting in swift intervention. Moreover, DECT can enhance vascular imaging, which is crucial for identifying vessel occlusions and determining treatment strategies like thrombectomy. Spectral CT goes beyond DECT by analyzing the energy-dependent attenuation of tissues. This technique allows for the enhanced visualization of subtle tissue changes associated with ischemic stroke, such as perfusion deficits and infarct expansion. Spectral CT’s material decomposition capabilities offer detailed information on tissue composition, which can help to distinguish penumbra (potentially salvageable tissue) from core infarction [1]. Photon-counting CT, a cutting-edge technology, directly detects individual X-ray photons and enables energy-resolved imaging. This enables exquisite tissue differentiation, which is critical in stroke cases where tissue distinctions are subtle. It can improve the accuracy of perfusion imaging and provide insights into collateral circulation, which are both essential for making stroke management decisions.

In summary, these advanced CT technologies offer remarkable potential to transform the imaging triage of acute ischemic stroke. They enhance tissue differentiation, aid in stroke subtype classification, refine perfusion assessments, and ultimately facilitate quicker and more tailored interventions, leading to improved patient outcomes and reduced morbidity.

### 2.2. Perfusion Imaging and Automated Imaging Platforms

The assessment of cerebral hemodynamics has gained prominence in stroke diagnosis. Techniques like arterial spin labeling (ASL) perfusion MRI provide non-invasive measurements of cerebral blood flow, aiding in the identification of compromised perfusion territories. Dynamic susceptibility contrast (DSC) and dynamic contrast-enhanced (DCE) perfusion MRI offer insights into hemodynamic parameters, allowing for the differentiation between penumbra and infarct core.

Perfusion imaging and automated imaging platforms may enhance the accuracy of stroke diagnostics. Perfusion imaging provides dynamic information about the blood flow within the brain, enabling the identification of salvageable tissue (penumbra) and core infarction. This knowledge is crucial for guiding time-sensitive decisions like thrombolysis or thrombectomy, especially in subgroups with borderline indications for reperfusion therapy. Perfusion imaging also allows for clinicians to not only assess tissue viability and match treatment options to individual patients, but also to predict recovery potential more accurately. This information is important to anticipate the patients’ courses and inform their family members. Automated platforms that are already used in daily clinical practice streamline the analysis of complex imaging data. These platforms rapidly process images to quantify perfusion deficits and penumbra volumes. This automation reduces inter-operator variability and time, allowing for clinicians to make timely decisions based on objectives and standardized data. Moreover, these platforms can be integrated into telestroke networks, facilitating expert consultation for remote hospitals that do not have stroke specialists onsite.

### 2.3. Machine Learning and Artificial Intelligence

Machine learning and artificial intelligence (AI) have brought about a paradigm shift in ischemic stroke diagnosis. These technologies enable the extraction of intricate patterns and insights from vast amounts of imaging and clinical data. AI algorithms can rapidly analyze images, identify subtle abnormalities, and quantify tissue characteristics, assisting radiologists in making accurate and timely diagnoses.

Deep learning models, when trained on large datasets, can detect small infarcts, assess lesion volume, and predict treatment responses. AI-driven software can also automatically segment brain structures and vessels, which saves time and reduces variability in radiological interpretations, leading to more standardized diagnoses.

### 2.4. Molecular Imaging and Biomarkers

Molecular imaging techniques offer a deeper understanding of ischemic stroke pathophysiology by visualizing molecular processes in real time. Positron emission tomography (PET) combined with radiotracers targeting specific molecular pathways can provide valuable information about inflammation, oxidative stress, and neurovascular remodeling in ischemic stroke cases.

CT densitometry-based methods used to directly quantify the net water uptake in the ischemic brain tissue have been increasingly applied in recent years [2]. There is potential for the net water uptake to be used as an imaging biomarker for the pathophysiology of infarcted lesions in clinical decision making. Artificial intelligence might help to address the lack of automation and standardization in the measurement of the net water uptake, which is an important current limitation.

### 2.5. Interventional Treatment

The use of thrombectomy in ischemic stroke cases has showcased remarkable advancements, expanding its application to patients with a large ischemic core, as supported by recent studies [3,4,5].

Currently running trials are investigating the effect of recanalization in distal vessel occlusions. Newer-generation stent retrievers and aspiration devices have demonstrated improved efficacy in reaching and removing clots lodged in smaller cerebral vessels. These innovations have widened the therapeutic window for endovascular treatment, enabling intervention in cases that were previously considered challenging.

Additionally, recent studies have investigated the efficacy of using thrombectomy to treat patients with a large ischemic core, where tissue damage was traditionally thought to be irreversible. Advanced imaging techniques, such as perfusion imaging, may help to identify salvageable tissue within the core, refining patient selection. Several trials have validated the benefit of thrombectomy even in these cases, showing improved functional outcomes when treatment is aligned with imaging-guided patient selection.

These breakthroughs underscore thrombectomy’s transformative potential in ischemic stroke care. By addressing distal occlusions and extending treatment to patients with substantial core infarctions, thrombectomy continues to push the boundaries of stroke intervention.

## 3. Challenges and Future Directions

While the advances in diagnostic radiology for ischemic stroke are promising, challenges remain. Access to advanced imaging technologies and expertise may be limited in certain regions, hindering their widespread adoption. Additionally, integrating these advanced techniques into routine clinical practice requires training and familiarity for radiologists and clinicians.

Further research is needed to validate the clinical utility of emerging techniques and refine their applications. Prospective studies are essential to establish the long-term benefits of advanced imaging in guiding treatment decisions, predicting outcomes, and enhancing patient care.

## 4. Studies of the Research Topic—Hemorrhagic Stroke

Cao et al. [6] assessed the first available automated 3D segmentation tool for intracerebral hemorrhage (ICH) based on a 3D neural network before and after retraining. Their proposed model showed a decent generalization in an external validation cohort, and the authors concluded that external validation and retraining are significant steps that need to be assessed before applying deep learning models in novel clinical settings.

It is known that ICH is associated with a significant long-term morbidity and a high mortality and therefore has a significant health economic impact. In particular, outcomes are poor if the onset is not known, but there are no established imaging-based tools that can be used to estimate the onset. Rusche et al. [7] assessed whether the onset estimation of ICH patients using AI may be more accurate than human readers. Interestingly, the diagnostic accuracy of AI-based classifiers for the onset determination of patients with ICH was low, suggesting that accurate AI-based onset assessment for patients with ICH based on CT data may not currently be likely to alter decision making in daily clinical practice. It was concluded that in the future, multimodal AI-based approaches might improve the precision of ICH onset prediction.

The precise detection of cerebral microbleeds (CMBs) using susceptibility-weighted (SWI) magnetic resonance imaging (MRI) is important for the characterization of several neurological diseases. Rusche et al. [8] evaluated the diagnostic performance of whole-body low-field MRI for the detection of CMBs in a prospective cohort of suspected stroke patients compared to an established 1.5 T MRI and observed that low-field MRI at 0.55 T might have a similar accuracy compared to 1.5 T scanners for the detection of CMBs, and thus may have great potential as an interesting alternative for future studies. In a further study, Rusche et al. [9] performed a detailed comparative analysis of low-field MRI including 27 ischemic stroke patients who all received 1.5 T and 0.55 T imaging. The authors discussed that low-field MRI with 0.55 T might not be inferior to MR scanners with higher field strengths, emphasizing their potential as low-cost alternatives in stroke imaging triage. Some limitations should nevertheless be considered, particularly with regard to the detection of very small infarcts.

## 5. Studies of the Research Topic—Ischemic Stroke

Perfusion imaging is often utilized to identify hypoperfusion in acute situations, but lacks availability in many stroke centers. Bunker et al. [10] discussed FLAIR hyperintense vessels (FHVs) in various vascular regions as an alternative method for quantifying hypoperfusion. In conjunction with prior work, their results support the use of FLAIR imaging to estimate the amount and location of hypoperfusion when perfusion imaging is not available.

In this context, it is important to note that the capability to precisely detect ischemic stroke and to predict neurological recovery at an early stage is of great clinical value. The study by Guo et al. [11] aimed to test the diagnostic performance of whole-brain dynamic radiomics features (DRF) for the detection of ischemic stroke and the assessment of neurological impairment. This study discussed an interesting clinical tool with a high potential for use in clinical practice to improve diagnosis and early outcome estimation even before treatment.

Another technique utilized in ischemic stroke triage is the perfusion-based hypoperfusion intensity ratio (HIR), which is known to be associated with collateral status, reflecting impaired microperfusion of the ischemic brain tissue in stroke patients presenting with a large vessel occlusion (AIS-LVO). Van Horn et al. [12] investigated whether HIR is directly associated with an early edema progression rate (EPR), which was assessed using the ischemic net water uptake (NWU) in a multicenter observational study of AIS-LVO patients treated via mechanical thrombectomy. An established quantitative imaging biomarker was utilized, and the authors discussed that a favorable HIR was directly linked to a lower EPR as measured on baseline NCCT.

In the past, it was observed that the formation of ischemic edema, which is the imaging hallmark of progressive infarction, may be directly influenced by thrombolytic treatment and endovascular recanalization. Broocks et al. [13] hypothesized that intravenous therapy with alteplase (IVT) is linked to a worse clinical outcome and increased ischemic edema formation when administered to ischemic stroke patients who subsequently achieved complete recanalization (defined as TICI 3) after MT. In summary, it was observed that bridging IVT was associated with an increased edema volume and risk of symptomatic intracerebral hemorrhage as secondary injury volumes. The results of this study encourage direct MT approaches, particularly in patients with a higher likelihood of successful recanalization.

After stroke, angiogenesis and neuroplasticity are important recovery processes. Recent advances in neuroimaging techniques might be used to assess these processes and become quantifiable indicators, or they may to be used to assess treatment effects. Wlodarczyk et al. [14] discussed neuroimaging techniques as potential tools to assess angiogenesis and neuroplasticity processes after ischemic stroke and discussed the potential clinical implications for recovery prognosis and rehabilitation.

As a further current important research topic, the detailed analysis of thrombi retrieved from endovascular procedures may be of great importance in order to increase the success of interventional treatment. Viltuznik et al. [15] investigated the accuracy of cerebral thrombus characterization via computed tomography (CT), magnetic resonance (MR), and histology, and how several parameters attained using these methods are correlated with each other. Finally, these parameters’ association with interventional procedures and clinical variables was investigated.

## 6. Conclusions

The field of diagnostic radiology has witnessed remarkable advances in the diagnosis of ischemic stroke, revolutionizing patient care and outcomes. Machine learning, advanced perfusion imaging, and quantitative imaging biomarkers have transformed our ability to detect and characterize ischemic stroke. These advances enable early intervention, accurate treatment decisions, and personalized care, ultimately leading to improved patient outcomes. As the momentum of innovation continues, collaborative efforts between clinicians, radiologists, and researchers will further drive the evolution of diagnostic radiology in the realm of ischemic stroke management.
